# Life-extending interventions do not necessarily result in compression of morbidity: a case example offering a robust statistical approach

**DOI:** 10.1007/s11357-025-01925-x

**Published:** 2025-11-12

**Authors:** Deependra K. Thapa, Wasiuddin Najam, Erik S. Parker, Xi Rita Wang, Daniel L. Smith, Ufuk Beyaztas, James F. Nelson, Steven N. Austad, Gary A. Churchill, David B. Allison

**Affiliations:** 1 Department of Epidemiology and Biostatistics, School of Public Health–Bloomington, Indiana University, Bloomington, IN USA; 2 USDA/ARS Children’s Nutrition Research Center, Baylor College of Medicine, Houston, TX USA; 3 Department of Nutrition Sciences, University of Alabama at Birmingham, Birmingham, AL USA; 4 Department of Statistics, Marmara University, Istanbul, Turkey; 5 Department of Cellular and Integrative Physiology, UT Health San Antonio, San Antonio, TX USA; 6 Department of Biology, University of Alabama at Birmingham, Birmingham, AL USA; 7 The Jackson Laboratory, Bar Harbor, ME USA

**Keywords:** Compression of morbidity, Healthspan, Lifespan, Lifespan-extending interventions, Survival, Vitality

## Abstract

Despite extensive research on life-extending interventions, rigorous statistical techniques to determine their impact on compression of morbidity (CoM) are rarely used. We present a case example of an analytical method for examining the effect of life-extending dietary interventions on CoM by comparing the rates of decline in vitality and survival toward the end of life. Using data from previous experimental studies in mice, we calculated the average rate of vitality decline by fitting exponential decay models to individual vitality trajectories and compared this rate with the rate of survival decline estimated from Cox proportional hazards model. The results showed that, surprisingly, the life-extending interventions failed to compress morbidity. Instead, the models suggested a potential expansion of morbidity with the interventions, particularly for chronic caloric restriction, as evidenced by a greater difference between the rates of vitality decline and survival decline in the intervention group than in the control group. The results further showed age-dependent effects, with interventions likely to demonstrate CoM at very old ages. These findings challenge the assumption that lifespan extension necessarily compresses morbidity, highlighting the need to consider lifespan, healthspan, and CoM as endpoints when evaluating anti-aging interventions. We do not claim that life-extending interventions categorically fail to achieve CoM; rather, we demonstrate how a rigorous statistical approach can be applied to test the CoM hypothesis. Our framework offers a valuable tool for future studies, and further refining this method will be crucial to determine under which circumstances lifespan extension leads to CoM. The potential to choose from multiple analyses of CoM calls for leadership in the geroscience community both to standardize the nomenclature so that different CoM analysis approaches can be communicated clearly and to establish a “default” CoM analysis for everyone to use in addition to their analyses of choice, thus facilitating comparisons and meta-analyses across studies.

**Supplementary Information:**

The online version contains supplementary material available at 10.1007/s11357-025-01925-x.

## Introduction

The compression of morbidity (CoM) hypothesis suggests that the proportion of life spent in a morbid state decreases, and individuals experience a greater proportion of their life in good health with a shorter period of morbidity, toward the end of life [1]. The hypothesis addresses how effectively health can be maintained as longevity increases and outlines two forms of morbidity compression: one in which morbidity rates decline more quickly than mortality rates, and the other in which the age of onset of chronic conditions rises faster than life expectancy [2]. Both forms of compression contribute to reducing cumulative lifetime morbidity, which ultimately improves population health. Conversely, morbidity is expanded when the proportion of life spent in suboptimal health rises [3].

During the past few decades, research has identified various interventions that can extend longevity in model organisms. These include caloric restrictions [4–6], mTOR inhibitory drugs such as rapamycin and rapalogs [7–9], drugs targeting insulin signaling pathways such as metformin [10, 11] and 17α-estradiol [12], glycemia regulation with glucosidase inhibitors such as acarbose [13], sodium glucose cotransporter 2 (SGLT2) inhibitors such as canagliflozin [14], NAD^+^-boosting drugs such as nicotinamide [15], senolytic treatments [16–19], spermidine [20, 21], chloroquine [22, 23], and epigenetic reprogramming strategies [24]. However, it remains largely unknown whether these interventions genuinely compress morbidity or simply prolong frailty and the duration of age-related diseases. On the one hand, a longevity intervention can compress morbidity by increasing years of healthy life, as proposed by Fries [1, 2]. On the other hand, the intervention could expand morbidity, by extending the period of illness. While the assumption that lifespan extension results in an increase in healthspan relative to increased lifespan, and thereby compresses morbidity [25], is intuitively appealing, it is infrequently examined. Previous research has shown that the global healthspan–lifespan gap has continued to widen. Over the past 20 years, the gap widened to 9.6 years worldwide, with the U.S. showing the largest disparity at 12.4 years [26]. This growing gap underscores the pressing challenges to achieving healthy longevity and highlights the urgent need for research to not only extend lifespan but address morbidity compression.

Some researchers studying human populations assert the existence of morbidity compression in the phase preceding death [27–30], whereas an opposing perspective suggests an expansion of the period marked by disability and ill health [31, 32]. Still others argue that the evidence is more nuanced and lacks a clear consensus [33–35]. There are some studies evaluating healthspan, lifespan, and CoM in animal models, the results of which are mixed. Teo and others [36], for instance, reported that a combination treatment of metformin and lithium extends healthspan but has no effect on lifespan in *Caenorhabditis elegans*, suggesting CoM. Colman and others [6] reported that caloric restriction delays the onset of chronic illnesses including diabetes, cancer, cardiovascular disease, and brain atrophy in rhesus monkeys and reduces the incidence of deaths associated with these diseases. Mattison et al. [37] also observed health benefits with caloric restriction in separate study of rhesus monkeys, albeit with no accompanying survival benefit observed. Hence, the latter study possibly demonstrated CoM, whereas the former did not necessarily. The exceptional longevity of Rottweiler dogs has also been found to be accompanied by cancer resistance and delayed onset of major diseases [38]. However, other studies in *C. elegans* [39, 40] and medflies [41] suggest that many interventions that extend lifespan also proportionally extend the period of illness, resulting in a greater proportion of life spent in a frail state. Thus, such interventions may not compress but instead expand morbidity.

It remains unclear whether these interventions extend healthspan proportionally to the lifespan, thereby compressing morbidity. There are several reasons for the lack of agreement in studies, including variation in morbidity measurements, data sources, analysis methods, characteristics of study subjects and participants, and place and time of data collection [42]. A critical gap remains in the availability of quantitative measures for assessing healthspan and statistically valid methods for examining CoM [43]. While the need to quantitatively measure healthspan and compare its extension relative to lifespan is compelling, there is no standard method to integrate longitudinal healthspan and lifespan measurements, and thereby examine CoM, in geroscience research.

Most studies in model organisms tend to analyze lifespan and healthspan outcomes separately, using distinct statistical models and significance thresholds. For example, researchers often compare survival curves using log-rank tests and evaluate frailty or health metrics using longitudinal models or summary scores, and then draw conclusions based on comparisons of *P*-values. This disjointed approach can lead to what is known as the differences in nominal significance (DINS) error—drawing incorrect inferences from differences in nonsignificance [44]. Furthermore, such analysis cannot determine the improvement in health(span) relative to lifespan, and hence cannot truly test CoM.

Some efforts have been made to address this gap. For instance, Yang et al. [45] proposed that interventions leading to a steeper survival curve may compress morbidity. In contrast, interventions that extend longevity while maintaining the original shape of the survival curve—a phenomenon referred to as scaling—are expected to proportionally prolong morbidity, without achieving compression. Lamming [46] introduced the summary measures FAMY (frailty-adjusted mouse years) and GRAIL (gauging robust aging when increasing lifespan) for quantifying healthspan more explicitly in mice. FAMY and GRAIL are conceptually similar to the concept of quality-adjusted life years (QALY) in humans. These composite metrics integrate lifespan and frailty index data to estimate the proportion of life lived in a healthy state. Similarly, Woo et al. [47] and Yu et al. [48] in epidemiological studies in humans have employed the ratio of sickspan to lifespan, or the ratio of frailty index to life expectancy, as indirect markers of morbidity compression, with a lower ratio implying greater degree of morbidity compression.

Despite their contributions, these approaches have important limitations. Comparing lifespan and healthspan separately fails to capture their joint relationship and often obscures whether health gains are proportional to life extension. Methods that dichotomize continuous health measures, such as frailty or vitality, into arbitrary morbidity thresholds can yield results that are sensitive to threshold selection, thereby compromising both validity and interpretability. Methods focusing on survival curve steepness can also mislead; for example, increasing lifespan without a concurrent delay in morbidity onset can steepen the curve while prolonging the sickspan. Composite measures like FAMY and GRAIL rely on the assumption that all animals begin the study with no frailty (frailty index = 0), which is rarely true in geroscience research, where animals are often enrolled in midlife. Moreover, censoring and early sacrifice of animals for tissue analysis lead to incomplete frailty and survival data, limiting the utility of these metrics.

**Fig. 1:**
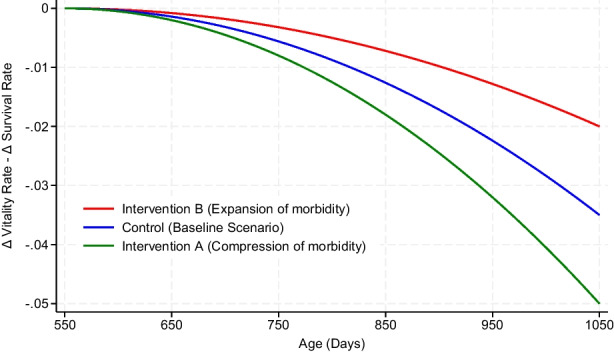
Hypothetical effect of life-extending intervention on compression (or expansion) of morbidity. Δ refers to the rate of change. Intervention A shows a slower decline in vitality relative to survival, suggesting compression of morbidity, while intervention B shows a faster decline in vitality than survival, indicating expansion of morbidity, both compared to control

The limited existing approaches and methods to examine CoM either rely on oversimplified assumptions, lack compatibility with longitudinal data structures, or require arbitrary choices that compromise reproducibility. There is a lack of rigorous statistical techniques to analyze whether longevity interventions also compress morbidity. This lack of statistical technique has hindered the progress in identifying interventions that effectively extend both lifespan and healthspan. This highlights an urgent need for statistically rigorous, interpretable, and replicable frameworks to directly test whether lifespan-extending interventions compress morbidity. In this paper, we illustrate a statistical approach to test whether interventions aimed at extending lifespan also achieve CoM. We propose that the CoM hypothesis can be evaluated by comparing the rate of health (vitality) decline with the rate of survival decline. As population age, both mortality and morbidity increase—that is, both survival and healthspan decline. Life-extending interventions aim to improve late-life survival by slowing the rate of decline in survival (i.e., slowing the rate of mortality). In addition to prolonging lifespan, such interventions may also affect healthspan, potentially slowing the rate of decline in health. We define CoM as a situation where the rate of decline in vitality (a measure of healthspan) is slower relative to the rate of decline in survival. Figure 1 shows a hypothetical effect of life-extending intervention on compression (or expansion) of morbidity.

Specifically, we assessed whether the first derivative of intraindividual vitality curves, reflecting the rate of vitality decline, is slower than the first derivative of the population survival curve, which reflects the rate of survival decline. We temporarily refer to this analytical approach as the *difference in rate effects (DIRE)* method. We hypothesized that the difference between the rate of decline in vitality and the rate of decline in survival is lower in the intervention group than in the control group, indicating that life-extending interventions not only extend lifespan but also compress morbidity. By operationalizing a quantitative framework that can be used, this study addressed the methodologic gap for evaluating whether lifespan-extending interventions also impact CoM.

## Methods

### Data sources

We utilized publicly available animal model data from Di Francesco et al. [49], Green et al. [50], and Shahmirzadi et al. [51], and did not collect primary data as part of this study. The details on study protocols, preregistration, study design, sample collection, and data processing, as well as relevant ethical approval, are documented in the original studies. These studies used mouse models to examine the effects of dietary interventions—including chronic caloric restriction, intermittent fasting, amino acid modulation, and alpha-ketoglutarate supplementation—on healthspan and lifespan. Table 1 summarizes the details of the studies including mouse strain, sex distribution, dietary comparison groups, group assignment methods, and age at the onset of intervention. Our secondary analysis using the data from these studies was not preregistered. However, the hypothesis—that the difference between the rate of decline in vitality and the rate of decline in survival is lower in the intervention group than in the control group—was developed a priori, before any data analyses were begun.

**Table 1:** Summary of animal studies used in the present analysis. The sample sizes presented represent those included in the present analysis. These may differ slightly from the total samples reported in the original studies due to variations in data availability and reporting. Shahmirzadi et al. [51] also included a second cohort; however, data from this cohort were unavailable for inclusion in the present analysis

Study	Mice strain	Sex	Comparison groups	Group assignment	Age at the start of intervention
Di Francesco et al. (2024) [49]	Genetically Diverse Outbred (DRiDO)	Female (*n* = 937)	5 groups: ad libitum feeding control (AL, *n* = 188); intermittent fasting 1 day per week (1D-IF, *n* = 188); intermittent fasting 2 consecutive days per week (2D-IF, *n* = 190); chronic caloric restriction at 20% (20% CR, *n* = 189); and chronic caloric restriction at 40% (40% CR, *n* = 182)	Random	6 months
Green et al. (2023) [50]	Genetically heterogeneous adult UM-HET3	Female (*n* = 89) and male (*n* = 92)	3 groups: 22% amino acid diet (Control AA, *n* = 31 female, 29 male); 7% amino acid diet (Low AA, *n* = 28 female, 33 male); and 22% amino acid diet with isoleucine reduced by 2/3rds (Low Ile, *n* = 30 female, 30 male)	Random	6 months
Shahmirzadi et al. (2020) [51]	C57BL/6	Female (*n* = 44) and male (*n* = 46)	2 groups: standard mouse diet feeding control (Control, *n* = 24 female, 24 male) and 2% w/w alpha-ketoglutarate supplementation (AKG, *n* = 20 female, 22 male)	Not clear	18 months

### Measurements

#### Frailty/vitality

To assess morbidity, these studies used a clinically relevant frailty index, as described by Parks et al. [52] and Whitehead et al. [53]. The frailty index comprised 31 phenotypes that serve as indicators of age-associated health deterioration across key physiological systems, including the musculoskeletal, vestibulocochlear/auditory, ocular, nasal, digestive, urogenital, and respiratory systems. Additional assessments included signs of discomfort, body weight, and body temperature. The frailty assessments were based on noninvasive techniques; hence, this type of manual handling would not be expected to impact survival, thus minimizing the risk of the assessment influencing survival outcomes. In a blinded manner, each phenotype was scored as 0, 0.5, or 1 on the basis of the severity of the deficit. A score of 0 indicated no sign of frailty for that phenotype, 0.5 reflected moderate frailty, and 1 represented severe frailty. The mean frailty index was then calculated as the sum of the individual trait scores divided by the total number of traits to generate a normalized score between 0 and 1, providing a comprehensive measure of age-related deficits and overall health deterioration. Green et al. [50] used only 28 of 31 traits to calculate the frailty index.

In the study by Di Francesco et al. [49], baseline frailty measurements were taken at 5 months of age, with subsequent assessments conducted approximately every 6 months, resulting in a total of six measurement points. For Green et al. [50], baseline assessments were conducted at 16 months, with follow-up every 2 months, resulting in a total of 11 data points. Shahmirzadi et al. [51] conducted baseline assessments at 18 months and repeated measurements every 8 weeks, yielding eight measurement points for the male groups and seven for the female groups.

#### Survival

The primary endpoint of these lifespan studies was natural death. The ages at which the mice were either found dead or selected for euthanasia were recorded. Euthanasia was performed for mice that were deemed unlikely to survive the next 48 h and were in substantial discomfort, following established guidelines. In the study by Di Francesco et al. [49], the median survival duration for mice was 931 days (range, 449 to 1638). In Green et al. [50], the median survival was 822 days for females (range, 527 to 1172) and 884 days for males (range, 563 to 1258). For Shahmirzadi et al. [51], the median survival duration was 891 days (range, 653 to 1063) for females and 994 days (range, 770 to 1207) for males.

### Statistical analyses

#### Calculation of vitality indices

At each measurement time point, a mean frailty index score (with a range of 0 to1), representing the average of the total frailty phenotypes, was calculated for each animal. This score served as a proxy for the animal’s overall health status, with a higher score indicating the presence of impairment, which is reflective of an animal’s increased vulnerability to adverse health outcomes, analogous to the concept of morbidity [54]. Given that we were interested in changes in vitality, the complement of frailty (i.e., 1 – frailty index) was calculated and referred to as *vitality*. Furthermore, for each subsequent time point, vitality was calculated as a proportion of the value recorded at the first measurement, and this proportional vitality measure was considered as the outcome in this analysis.

#### Conceptual curves and their first derivatives

To test the CoM hypothesis, we plotted individual trajectories of vitality and fitted models to these curves to assess changes in health status and compared the rate of decline in vitality with the rate of decline in survival. For CoM, the rate of decline in vitality should be less than the rate of decline in survival.

The intraindividual vitality curves, reflecting how an individual’s vitality changes with age relative to baseline, were modeled via an exponential decay function as follows:

$${v}_{t}={v}_{0}*{e}^{-\lambda *t}+c$$

where:

- ${v}_{t}$ is the vitality at age $t$,
- ${v}_{0}$ is the baseline vitality at $t$ = 0 (set as 1),
- $\lambda$ is the exponential decay rate,
- $t$ is the age (in days), and
- $c$ is the asymptotic vitality value (set as the minimum value of vitality observed for that mouse).

From this model, the first derivative of the vitality curve (*dV*) for each individual mouse, which is the rate of change in vitality at specific time points, is calculated as follows:

$$\frac{d{v}_{t}}{dt}={v}_{0}\lambda *{e}^{-\lambda *t}$$

The rate of change in vitality was estimated for each individual mouse starting at 18 months of age (540 days, roughly equivalent to 60 human years), and subsequently at 30-day intervals, as well as on the actual frailty assessment days. Analyses were restricted to mice that were alive at each specific time point. The average rate of change in vitality across all mice was calculated based on intraindividual rates of change using the 30-day interval data (see Supplementary Tables 4 to 8) and the actual frailty measurement days (see Supplementary Tables 9 to 13).

The survival function *S*(t) was estimated via the Cox proportional hazards model as follows:

$$S\left(t|X\right)= {\left[{S}_{0}\left(t\right)\right]}^{e\left(\beta *X\right)}$$

where:

- $S\left(t|X\right)$ is the survival function at time *t* given treatment *X*,
- ${S}_{0}\left(t\right)$ is the baseline survival function, and
- *β* is the regression coefficient for treatment *X*, representing the effect of the treatment on survival.

From this model, the first derivative of survival function (*dS*), representing the rate of change in survival over time, was calculated at the same ages (in days) at which the rate of change in vitality was estimated. This was done separately for the treatment and control groups, as follows.

$$\frac{dS}{dt}=\frac{{S}_{(ti+1)}-{S}_{ti}}{{t}_{i+1}-{t}_{i}}$$

where:

- ${t}_{i}$ is the current time point,
- ${t}_{i+1}$ is the next time point,
- ${S}_{ti}$ is the survival probability at ${t}_{i}$, and
- ${S}_{(ti+1)}$) is the survival probability at ${t}_{i+1}$.

#### Comparison of rates of change in vitality and survival

The estimated rates of change in vitality and survival were negative at all time points, indicating a decline in both vitality and survival as the mice aged. For further analysis, we used the absolute values of the rates (|*dV*| for vitality and |*dS*| for survival) and interpreted them as the rates of decline in vitality and survival, respectively. For within-group (difference) analysis, the rates of decline in vitality and survival were compared using paired *t*-tests and the Wilcoxon signed-rank test. For between-group (difference-in-differences) analysis, the differences between the rates of decline in vitality and survival were calculated, and the means of these difference scores were compared using one-way ANOVA, the Kruskal‒Wallis test, and linear regression. In the case of Shahmirzadi et al. [51], between-group analyses were conducted using independent sample *t*-test and Wilcoxon rank-sum test, as there were only two comparison groups in this study. Standard errors and *P*-values were further obtained through bootstrapping with 1000 resamples. We employed multiple tests to ensure robustness across different distributional assumptions; parametric tests provided sensitivity under normality, while nonparametric counterparts offered reliability when assumptions were violated. Bootstrapping further enhanced statistical rigor by providing stable estimates of standard errors and *P*-values, particularly important given the small sample size. Statistical analysis was performed via Stata version 18.0. All tests were two-sided, with *P*-values < 0.05 considered statistically significant.

## Results

### Change in vitality

**Fig. 2:**
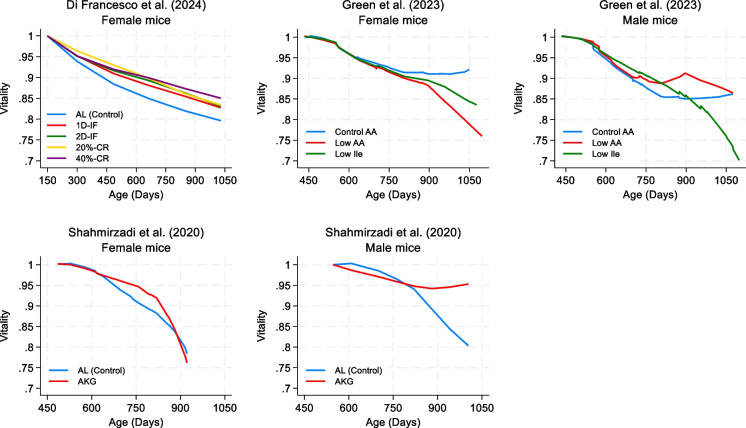
Mean vitality trajectories by treatment group across different ages. The vitality plotted in the figure is the proportion of the value recorded at the first measurement. See Table 1 for additional experimental details. Abbreviations: AA, amino acid; AL, ad libitum; AKG, alpha-ketoglutarate supplementation; 20%-CR, caloric restriction at 20%; 40%-CR, caloric restriction at 40%; Ile, isoleucine; 1D-IF, intermittent fasting 1 day per week; 2D-IF, intermittent fasting 2 consecutive days per week

Figure 2 presents the vitality trajectories of mice at different ages by treatment group. As the mice aged, vitality declined in all studies, although the rate and extent of decline varied between groups and across studies.

### Survival analysis

**Fig. 3:**
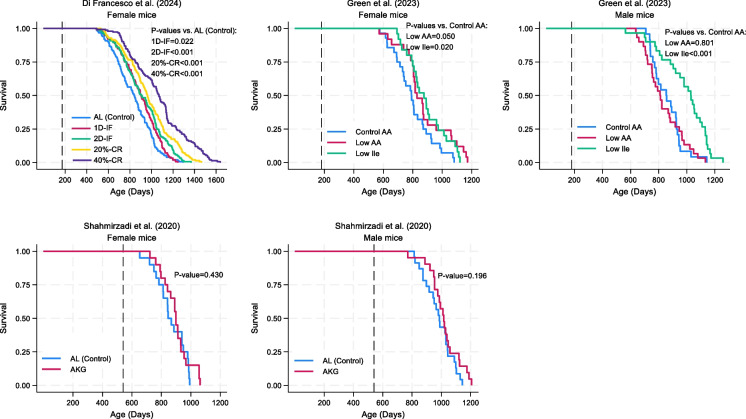
Kaplan–Meier survival curves by intervention groups. The vertical dashed lines represent the initiation point of the intervention. See Table 1 for additional experimental details. Abbreviations: AA, amino acid; AL, ad libitum; AKG, alpha-ketoglutarate supplementation; 20%-CR, caloric restriction at 20%; 40%-CR, caloric restriction at 40%; Ile, isoleucine; 1D-IF, intermittent fasting 1 day per week; 2D-IF, intermittent fasting 2 consecutive days per week

The Kaplan–Meier survival curves showed that all interventions in the study by Di Francesco et al. [49] significantly extended lifespan compared with the control group. Among the intervention groups, the effect was most pronounced in the 40% caloric restriction group, followed by the 20% caloric restriction group, the group assigned to intermittent fasting 2 consecutive days per week, and the group assigned to intermittent fasting 1 day per week. In the study by Green et al. [50], dietary restriction of isoleucine significantly improved survival compared with the control group in both male and female mice. No significant survival benefits were observed with 2% w/w alpha-ketoglutarate supplementation in either sex in the case of Shahmirzadi et al. [51] (Fig. 3).

.

In the following sections, we present the results comparing the rates of decline in vitality and survival. We begin with the within-group (difference) analysis, followed by the between-group (difference-in-differences) analysis. The analyses shown here are based on rates of decline estimated at 30-day intervals starting from 540 days of age. Results based on actual frailty measurement days are provided in Supplementary Tables 1 and 2.

### Rates of decline in vitality and survival: difference analysis

Table 2 presents the differences between the rates of decline in vitality (|*dV*|) and survival (|*dS*|) within each intervention group. Across all studies in both sexes, the rate of decline in vitality was consistently lower than the rate of decline in survival in all groups. The results were robust across different statistical methods, including paired *t*-tests, bootstrapped paired *t*-tests, and nonparametric Wilcoxon signed-rank tests. Similar patterns were observed when the analysis was conducted using the rates of decline in vitality and survival on actual frailty assessment days (Supplementary Table 1), although some variation in statistical significance was observed. In Di Francesco et al. [49], the differences were not statistically significant in any of the groups. In Green et al. [50], significant differences were found in all three groups for female mice, but only in the 7% amino acid diet (Low AA) group for male mice. In the case of Shahmirzadi et al. [51], female mice exhibited significant differences between the rates of decline in vitality and survival in only the intervention (alpha-ketoglutarate [AKG] supplementation) group, whereas the differences were not significant in male mice in either group (Supplementary Table 1).

**Table 2:** Differences between rates of decline in vitality and survival, by intervention group (within-group analysis)

Treatment	Variables	n*	Mean	Paired *t*-test					Wilcoxon signed-rank test	
				**Mean difference (vitality–survival)**	**SE**	***P*-value**	**Bootstrapped SE**	**Bootstrapped *P*-value**	***P*-value**	**Bootstrapped *P*-value**
**Di Francesco et al. **[49]**—female mice**										
AL (Control)	Rate of decline in vitality	25	0.00015	− 0.00107	0.00010	< 0.001	0.00009	< 0.001	< 0.001	< 0.001
	Rate of decline in survival	25	0.00122							
1D IF	Rate of decline in vitality	23	0.00012	− 0.00106	0.00009	< 0.001	0.00009	< 0.001	< 0.001	< 0.001
	Rate of decline in survival	23	0.00118							
2D IF	Rate of decline in vitality	27	0.00012	− 0.00094	0.00008	< 0.001	0.00008	< 0.001	< 0.001	< 0.001
	Rate of decline in survival	27	0.00105							
20% CR	Rate of decline in vitality	30	0.00013	− 0.00078	0.00007	< 0.001	0.00007	< 0.001	< 0.001	< 0.001
	Rate of decline in survival	30	0.00091							
40% CR	Rate of decline in vitality	34	0.00011	− 0.00068	0.00007	< 0.001	0.00007	< 0.001	< 0.001	< 0.001
	Rate of decline in survival	34	0.00079							
**Green et al. **[50]**—female mice**										
Control AA	Rate of decline in vitality	18	0.00021	− 0.00143	0.00032	< 0.001	0.00031	< 0.001	< 0.001	< 0.001
	Rate of decline in survival	18	0.00164							
Low AA	Rate of decline in vitality	21	0.00029	− 0.00113	0.00022	< 0.001	0.00021	< 0.001	< 0.001	< 0.001
	Rate of decline in survival	21	0.00142							
Low Ile	Rate of decline in vitality	19	0.00027	− 0.00111	0.00024	< 0.001	0.00023	< 0.001	< 0.001	< 0.001
	Rate of decline in survival	19	0.00138							
**Green et al. **[50]** – male mice**										
Control AA	Rate of decline in vitality	20	0.00033	− 0.00120	0.00023	< 0.001	0.00023	< 0.001	< 0.001	< 0.001
	Rate of decline in survival	20	0.00153							
Low AA	Rate of decline in vitality	19	0.00022	− 0.00136	0.00025	< 0.001	0.00025	< 0.001	< 0.001	< 0.001
	Rate of decline in survival	19	0.00158							
Low Ile	Rate of decline in vitality	22	0.00033	− 0.00088	0.00023	0.001	0.00023	< 0.001	< 0.001	< 0.001
	Rate of decline in survival	22	0.00121							
**Shahmirzadi et al. **[51]**—female mice**										
Control	Rate of decline in vitality	15	0.00060	− 0.00140	0.00047	0.010	0.00045	0.002	0.015	< 0.001
	Rate of decline in survival	15	0.00200							
Treatment	Rate of decline in vitality	17	0.00024	− 0.00145	0.00043	0.004	0.00041	< 0.001	0.002	< 0.001
	Rate of decline in survival	17	0.00169							
**Shahmirzadi et al. **[51]**—male mice**										
Control	Rate of decline in vitality	20	0.00041	− 0.00111	0.00041	0.014	0.00041	0.006	0.022	0.006
	Rate of decline in survival	20	0.00152							
Treatment	Rate of decline in vitality	22	0.00013	− 0.00126	0.00037	0.003	0.00035	< 0.001	0.002	< 0.001
	Rate of decline in survival	22	0.00139							

^*^n refers to the number of measurement points (ages) at which vitality and survival were estimated

*AA* amino acid, *AL* ad libitum, *20% CR* caloric restriction at 20%, *40% CR* caloric restriction at 40%, *Ile* isoleucine, *1D IF* intermittent fasting 1 day per week, *2D IF* intermittent fasting 2 consecutive days per week

### Rates of decline in vitality and survival: difference-in-differences analysis

When comparing the differences in the rates of decline in vitality and survival between the treatment groups and the control group, we found that the results varied across the studies. In the case of Di Francesco et al. [49], the lowest difference was observed in the control, with the difference-in-differences scores statistically significant for the 20% caloric restriction and 40% caloric restriction groups, suggesting that the chronic caloric restriction contributed to expansion of morbidity, which is opposite to what we hypothesized. In Green et al. [50], the lowest difference in female mice was observed in the control group, whereas in male mice, the lowest difference was in the Low AA group. In Shahmirzadi et al. [51], the treatment (AKG supplementation) group showed lower difference-in-differences scores compared with the control in both female and male mice. However, these differences were not statistically significant in Green et al. [50] and Shahmirzadi et al. [51] (Table 3).

**Table 3:** Difference-in-differences (between-group) analysis of the rate of decline in vitality and survival

Sample	Group	n*	Mean difference (vitality–survival)	One-way ANOVA		Kruskal‒Wallis		Linear regression			
				**F statistic and *P*-value**	**Bootstrapped *P*-value**	***χ***^**2**^	***P*-value**	**Difference-in-differences (treatment–control)**	**SE of the mean difference**	***P*-value**	**Bootstrapped *P*-value**
**Di Francesco et al. **[49]											
Female	Control (AL)	25	− 0.00107	*F* = 4.56, *P* = 0.002	0.095	17.52	0.002	Ref			
	1D IF	23	− 0.00106					0.00002	0.00013	0.901	0.902
	2D IF	27	− 0.00094					0.00014	0.00012	0.275	0.279
	20% CR	30	− 0.00078					0.00029	0.00012	0.016	0.013
	40% CR	34	− 0.00068					0.00039	0.00012	0.001	0.001
**Green et al. **[50]											
Female	Control AA	18	− 0.00143	*F* = 0.46, *P* = 0.631	0.745	0.74	0.693	Ref			
	Low AA	21	− 0.00113					0.00030	0.00039	0.436	0.435
	Low Ile	19	− 0.00111					0.00032	0.00040	0.425	0.408
Male	Control AA	20	− 0.00120	*F* = 1.08, *P* = 0.348	0.656	3.29	0.193	Ref			
	Low AA	19	− 0.00136					− 0.00016	0.00034	0.643	0.633
	Low Ile	22	− 0.00088					0.00032	0.00033	0.332	0.339
**Shahmirzadi et al. **[51]											
				**Independent sample *t-*test**					**Wilcoxon rank-sum test**		
			**Mean (difference)**	**Difference-in-differences**	**SE**	***P*-value**	**Bootstrapped SE**	**Bootstrapped *P*-value**	***P*-value**	**Bootstrapped *P*-value**	
Female	Treatment	17	− 0.00145	− 0.00005	0.00063	0.933	0.00066	0.935	0.823	0.823	
	Control	15	− 0.00140								
Male	Treatment	22	− 0.00126	− 0.00015	0.00055	0.780	0.00055	0.780	0.266	0.273	
	Control	20	− 0.00111								

^*^n refers to the number of measurement points (ages) at which vitality and survival were estimated

*Abbreviations*: *AA* amino acid, *AL* ad libitum, *20% CR* caloric restriction at 20%, *40% CR* caloric restriction at 40%, *Ile* isoleucine, *1D IF* intermittent fasting 1 day per week, *2D IF* intermittent fasting 2 consecutive days per week

When analyzed using the rates of decline in vitality and survival using the data on the actual frailty assessment days (Supplementary Table 2), we found no significant differences in any of the studies. In general, these results showed no evidence of greater morbidity compression due to the intervention.

**Fig. 4:**
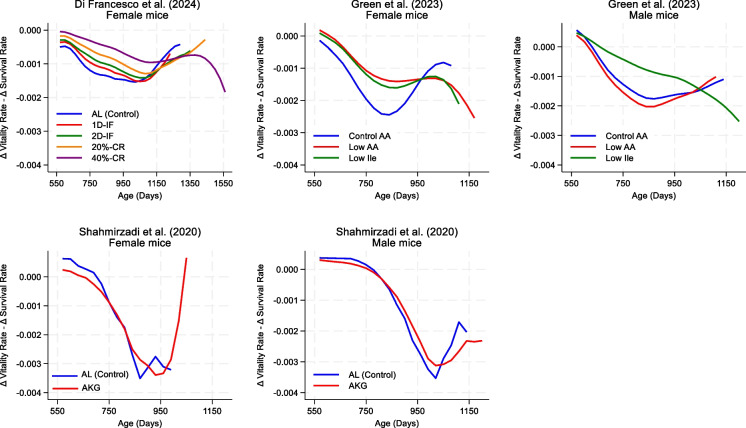
Differences between rates of decline in vitality and survival by treatment group across different ages. Abbreviations: AA, amino acid; AL, ad libitum; AKG, alpha-ketoglutarate supplementation; 20%-CR, caloric restriction at 20%; 40%-CR, caloric restriction at 40%; Ile, isoleucine; 1D-IF, intermit-tent fasting 1 day per week; 2D-IF, intermittent fasting 2 consecutive days per week

In Fig. 4, we plotted the scores for the differences between the rates of vitality and survival across age and treatment groups. Negative values in all cases indicate that vitality declined more slowly than survival in all comparison groups.

Older mice in the control group, in general, showed lower differences between vitality and survival rates compared with the intervention groups. However, this pattern appeared to reverse in very old (late life) mice, with the intervention groups showing larger negative differences compared with the control, indicating a CoM in the intervention groups in very old mice. A separate analysis in this age range confirmed consistently lower differences in the intervention groups, except for male mice in Green et al. [50], for which the Low AA group had a higher difference than the control. For female mice in Green et al. [50], bootstrapped results for the difference-in-differences showed statistical significances, suggesting that the Low AA group experienced greater morbidity compression than did the control group (Supplementary Table 3).

## Discussion

Given the lack of rigorous statistical methods for analyzing CoM, we have proposed an analytic approach to test whether interventions designed to extend lifespan also achieve CoM. Specifically, we evaluated the CoM hypothesis by comparing the rate of vitality decline with the rate of survival decline toward the end of life. This method, which we refer to as DIRE, involved assessing whether the average first derivative of intraindividual vitality curves, which reflects the rate of vitality decline, is slower than the first derivative of the population survival curve, which reflects the rate of survival decline.

The CoM hypothesis, proposed by James Fries in the 1980s [1, 2, 55], suggested that the onset of chronic diseases would progressively shift to older ages more rapidly than improvements in life expectancy, resulting in a shorter duration of morbidity compressed toward the end of life. This view was later challenged by the expansion of morbidity hypothesis articulated in 1991 [56], which argued that medical advances would extend lifespan without improving healthspan, thereby prolonging chronic illness. The conceptual roots of this debate trace back to Greunberg’s “failure of success” hypothesis proposed in 1977 [57], which suggested that reducing mortality from acute disease could increase survival while also raising the prevalence of chronic conditions. A critical assumption underlying these frameworks is that aging itself is unmodifiable. In contrast, our study directly evaluates morbidity and mortality dynamics under conditions where aging trajectories can be modifiable. By examining morbidity and mortality dynamics in a context of modifiable aging, our work extends prior theoretical formulations and offers a critical new perspective on this long-standing debate.

We found that the rate of decline in survival does not necessarily align with the decline in healthspan nor compress morbidity, suggesting that lifespan, healthspan, and CoM can be independent of each other. Ultimately, these interventions may prolong the frailty period, extending the duration of age-related health decline. Lifespan extension without corresponding CoM may not be an ideal strategy for aging populations, given the substantial financial and personal costs associated with age-related morbidity [58]. A finding that life-extending interventions failed to compress morbidity would have major implications for both preclinical research and clinical translation. Such a finding points to the importance of shifting focus from merely extending life to ensuring quality of those additional years of life and highlights the need for joint health–survival analyses like our DIRE method to inform future geroscience interventions. In the quest to achieve healthy aging, future geroscience will benefit from continuing its ongoing shift from an emphasis on merely prolonging life to also enhancing healthspan and compressing morbidity [39]. Lifespan may not be the best outcome measure if the aim is to identify interventions with meaningful benefits for human health, and future preclinical studies should place greater attention on health metrics to better understand how interventions modulate the aging process. Our findings challenge the current paradigm of research on aging which seems to assume CoM, and highlight the need to study healthspan alongside lifespan in the evaluation of aging-related interventions. We echo Scott [59], who calls for the next frontier in research on aging to focus on ensuring that longer lives are also healthier lives.

Our findings further suggest that life-extending interventions may have age-dependent effects on CoM. In particular, the observed reversal of the pattern in very old mice, for which intervention groups exhibited greater CoM, may indicate a shift in the impact of intervention in later life. However, this interpretation is constrained by limitations in the data. The number of mice surviving to very old age was low, and the separate analysis for this age group included fewer time points, potentially affecting the robustness of the results. Additionally, given the advanced age of the mice, it is likely that mice would die of old age in this cohort, raising the possibility that the apparent CoM reflects a more rapid decline in survival rather than a slower decline in vitality. Future studies should aim to address these limitations by including larger sample sizes and more frequent assessments of vitality, particularly in the later stages of life.

Our findings are consistent with previous studies suggesting that lifespan-extending interventions may not impact healthspan relative to lifespan. For instance, Harrison et al. [8] reported that while rapamycin treatment in mice increased the age at death, indicating an extension in maximal lifespan, the treated mice eventually suffered from the same types of diseases as the control group, primarily cancer and cardiovascular disease. Similarly, Corder et al. [60] examined the effects of interventions known to extend lifespan, such as 17α-estradiol and caloric restriction, on physiological resilience (which can be considered a measure of vitality) in mice. Interestingly, these interventions did not enhance resilience as expected. Our results align with previous research showing that lifespan and healthspan metrics capture different facets of aging in mice [25, 61], *C. elegans* [39], and nonhuman primates [6].

It is important to note that the apparent expansion of morbidity in the intervention groups might be due to a reduced mortality rate rather than an accelerated decline in vitality. This suggests that while the aging rate, at least for the caloric restriction group, may be modified, the period of (non) morbidity and lifespan are extended proportionally, leading to a linear time extension of morbidity corresponding to the extension of lifespan. While our approach focuses on the relationship between the rates of change in vitality and survival, complementary methods such as examining the slope of mortality curves or the age of onset and progression of disability conditions would provide additional insight, and should be further examined.

Our analysis evaluating CoM has some limitations. A potential issue in our analysis is the difference in how vitality and survival rates were calculated. Survival gives only one time point (death) per animal, necessitating group-level computation, whereas vitality rates were estimated at the individual level for animals alive at each time point and then averaged. While this approach allows for consistent tracking of vitality trajectories, it may lead to interpretive challenges because dead animals are excluded from subsequent evaluations, potentially reducing group morbidity scores. Furthermore, the two measures have different temporal resolutions: vitality was measured at more frequent intervals, whereas survival was tracked continuously. This mismatch in temporal resolution can overlook short-term morbidity changes that happen within weeks before death, like weight loss or temperature dips. Strain-specific responses to lifespan interventions are well-documented [62], and the findings reported here may therefore be specific to C57BL/6, DO, or HET3 mice. We also acknowledge that the lack of observed differences in CoM across the intervention groups could have been due to sample size limitations, as the ultimate sample size for testing differences was constrained by the number of measurement points. Additionally, mice were co-housed in groups of two to eight per cage in the included trials. Recent studies suggest that co-housed mice may exhibit correlated outcomes, violating the assumption of independence, and ignoring such clustering can lead to underestimated variance and biased results [63]. However, due to the lack of detailed co-housing information in the available datasets, we were unable to account for these potential clustering effects in our analysis.

Our analysis focused on data from three studies involving dietary interventions. Other life-extending interventions that influence aging through different mechanisms, such as pharmacological or genetic interventions, may have different effects. Future studies applying the proposed analytical methods to a broader range of life-extending interventions are needed to determine whether similar patterns of CoM—or lack thereof—are observed. The results of this analysis should be compared with previous studies examining the impact of interventions on healthspan relative to lifespan, particularly with Yang et al. [45], who proposed that interventions that steepen the survival curve can compress morbidity, and Lamming [46], who introduced FAMY and GRAIL as summary measures for quantifying healthspan in mice. Further studies should apply the proposed analytical approach to longitudinal aging studies in humans, integrating both morbidity and mortality measures to provide a more comprehensive understanding of CoM in human populations.

Another important consideration involves how “natural deaths” are defined and how these relate to intrinsic versus extrinsic causes of mortality [64]. It is possible that some natural deaths were influenced by extrinsic causes. Detailed pathological assessments were not performed for every animal, as this is outside the scope of most large-scale lifespan studies. For this reason, the investigators relied on time to natural death—defined as animals found dead or euthanized when near death—which is a standard and widely accepted proxy for death due to aging. Some natural deaths may result from extrinsic factors, but separating intrinsic and extrinsic mortality is challenging because it requires detailed assessment of the cause of death for each individual. We acknowledge this complexity and encourage researchers in this field to remain mindful of this possibility. In the studies considered here, however, extrinsic causes of mortality were unlikely to be a major issue, because experiments were conducted under pathogen-free conditions and staff were trained to minimize risks of infectious disease. With few exceptions, mice in these studies died due to failure to maintain homeostasis in one or more internal systems (e.g., cancers or kidney failure), which could reasonably be considered intrinsic aspects of the aging process.

One question of interest is how the distribution of longevity might relate to our DIRE method. It is well established that survival curves can follow different distributions: they are often approximated by the Gompertz distribution [65], but survival times can also follow Weibull, Gamma, or other distributions. An advantage of our DIRE method is that it can jointly analyze rates of decline in survival and vitality to test whether healthspan keeps pace with lifespan, regardless of the underlying distribution of longevity. However, it is possible that with different distributions, the method may perform somewhat differently and can affect certain aspects, such as the statistical power of the method. Exploring how longevity distributions influence DIRE results would be an interesting subject for future research.

When translating these findings to humans, it is important to recognize that human health and mortality are strongly shaped by cohort effects and secular trends [66]—such as differences in early-life conditions, medical care, diet, and lifestyle—which can influence mortality and morbidity dynamics independently of aging interventions. In contrast, preclinical studies are conducted in controlled environments that reduce such variability and allow clearer isolation of intervention effects, but they lack the complexity of human aging. Therefore, while our methodology provides a valuable framework for evaluating aging interventions, its application to human populations must be approached with explicit consideration of cohort and secular trends.

We have offered one approach by which CoM can be formally tested. The DIRE method we propose here, to the best of our knowledge, represents the first rigorous attempt to examine CoM by comparing both healthspan and lifespan using a comprehensive analytical approach. This method offers several advantages. First, our method allows for a formal statistical test of significance to examine the effect of an intervention on CoM. Second, it enables a simultaneous analysis of differences in both survival and vitality rates by treatment assignment—avoiding the common pitfall of conducting separate analyses of survival data and vitality data and comparing *P*-values, which can lead to the DINS error [44, 67]. Third, the method can flexibly accommodate varying time points across and within studies, as well as different measures of vitality. Fourth, there is no need to arbitrarily dichotomize a continuous measure of vitality (or health, ability, or function) into “morbidity” and “nonmorbidity” categories. Given these strengths, we believe that the DIRE method offers a uniform means of analysis, allowing comparability across studies.

An important consideration is that vitality in this study was entirely defined by frailty, and not by other healthspan assays or cellular and molecular hallmarks of aging. While our analytical approach is designed to accommodate any robust quantitative measure of healthspan or morbidity, such as health, function, or vitality, we used frailty (and its complement, vitality) due to its well-established use in the field and its longitudinal availability across the included datasets. We recognize that different investigators may make different choices in how to assess healthspan and quantify CoM, and those choices can lead to different results. For example, Dr. Dudley W. Lamming, who critically commented on the draft version of this manuscript, believes that the most direct way to assess this question is to measure, for each animal, both the absolute time and the proportion of lifespan spent above a predefined vitality threshold. This approach would estimate the fraction of life lived in good health and facilitate comparisons across interventions with differing survival patterns. While we did not implement this analysis here, we recognize its potential value and plan to address it in future work.

The DIRE method can serve as a default framework for statistical integration while remaining flexible to accommodate different healthspan indices. Of course, even if the DIRE method were adopted as a standard for all “apples-to-apples” comparisons across studies in the field, additional approaches could be used for sensitivity analyses. We propose that the potential to choose from multiple analyses of CoM calls for leadership in the geroscience community to (a) promote preregistration of data analytic plans; (b) standardize the nomenclature so that different CoM analysis approaches can be communicated clearly; and (c) establish a “default” CoM analysis for everyone to use in addition to their analyses of choice enabling apples-to-apples comparisons and meta-analyses across studies.

## Conclusion

Our findings challenge the assumption that lifespan extension necessarily results in CoM. In our rigorous statistical analysis, we found no evidence supporting the idea that lifespan-extending interventions lead to CoM. These findings underscore the importance of considering lifespan, healthspan, and CoM as endpoints when evaluating anti-aging interventions. While lifespan extension may increase the number of healthy years, it may also extend the frailty period, with substantial social and economic implications. We do not claim that lifespan-extending interventions categorically fail to compress morbidity. Rather, we illustrate here how a rigorous analytical approach can be applied to analyze the hypothesis of CoM. In the absence of robust and standardized methods for evaluating this relationship, our approach provides a useful framework for future studies. Further refining of this method will be crucial to determine under which circumstances lifespan extension leads to CoM.

## Supplementary Information

Below is the link to the electronic supplementary material.

Supplementary Material 1 (DOCX 42.0 KB)

Supplementary Material 1 (DOCX 42.0 KB)

Supplementary Material 2 (XLSX 50.8 KB)

Supplementary Material 2 (XLSX 50.8 KB)

## Data Availability

We utilized publicly available data from Di Francesco et al. [49], Green et al. [50], and Shahmirzadi et al. [51], and did not collect primary data as part of this study. The raw data used in this study can be accessed from FigShare (10.6084/m9.figshare.24600255), and Mendeley (https://data.mendeley.com/datasets/dk74jscsww/3 and 10.17632/zr4ffhd3k9.1). The processed data used in the analysis are included in Supplementary Tables 4 to 13.

## Code availability

Codes used in our analysis are available at https://osf.io/dezrh.

## Declarations

### Disclaimer

The assertions expressed are those of the authors and not necessarily those of the NIH or any other organization.

## References

[1] Fries JF. The compression of morbidity: near or far? Milbank Q. 1989;67(2):208–32. 10.2307/3350138. 2698444

[2] Fries JF. The compression of morbidity. Milbank Q. 1983;83(4):801–23. 10.1111/j.1468-0009.2005.00401.x. 10.1111/j.1468-0009.2005.00401.x PMC2690269 16279968

[3] Manuel DG, Schultz SE, Kopec JA. Measuring the health burden of chronic disease and injury using health adjusted life expectancy and the health utilities index. J Epidemiol Community Health. 2002;56(11):843–50. 10.1136/jech.56.11.843. 12388577 10.1136/jech.56.11.843 PMC1732044

[4] Yamada Y, Kemnitz JW, Weindruch R, Anderson RM, Schoeller DA, Colman RJ. Caloric restriction and healthy life span: frail phenotype of nonhuman primates in the Wisconsin National Primate Research Center Caloric Restriction Study. J Gerontol A Biol Sci Med Sci. 2017;73(3):273–8. 10.1093/gerona/glx059. 10.1093/gerona/glx059 PMC5861888 28398464

[5] Mattison JA, Colman RJ, Beasley TM, Allison DB, Kemnitz JW, Roth GS, et al. Caloric restriction improves health and survival of rhesus monkeys. Nat Commun. 2017;8(1):14063. 10.1038/ncomms14063. 28094793 10.1038/ncomms14063 PMC5247583

[6] Colman RJ, Anderson RM, Johnson SC, Kastman EK, Kosmatka KJ, Beasley TM, et al. Caloric restriction delays disease onset and mortality in rhesus monkeys. Science. 2009;325(5937):201–4. 10.1126/science.1173635. 19590001 10.1126/science.1173635 PMC2812811

[7] Robida-Stubbs S, Glover-Cutter K, Lamming DW, Mizunuma M, Narasimhan SD, Neumann-Haefelin E, et al. TOR signaling and rapamycin influence longevity by regulating SKN-1/Nrf and DAF-16/FoxO. Cell Metab. 2012;15(5):713–24. 10.1016/j.cmet.2012.04.007. 22560223 10.1016/j.cmet.2012.04.007 PMC3348514

[8] Harrison DE, Strong R, Sharp ZD, Nelson JF, Astle CM, Flurkey K, et al. Rapamycin fed late in life extends lifespan in genetically heterogeneous mice. Nature. 2009;460(7253):392–5. 10.1038/nature08221. 19587680 10.1038/nature08221 PMC2786175

[9] Bitto A, Ito TK, Pineda VV, LeTexier NJ, Huang HZ, Sutlief E, et al. Transient rapamycin treatment can increase lifespan and healthspan in middle-aged mice. Elife. 2016;5:e16351. 10.7554/eLife.16351. 27549339 10.7554/eLife.16351 PMC4996648

[10] Alfaras I, Mitchell SJ, Mora H, Lugo DR, Warren A, Navas-Enamorado I, et al. Health benefits of late-onset metformin treatment every other week in mice. NPJ Aging Mech Dis. 2017;3(1):16. 10.1038/s41514-017-0018-7. 29167747 10.1038/s41514-017-0018-7 PMC5696465

[11] Martin-Montalvo A, Mercken EM, Mitchell SJ, Palacios HH, Mote PL, Scheibye-Knudsen M, et al. Metformin improves healthspan and lifespan in mice. Nat Commun. 2013;4:2192. 10.1038/ncomms3192. 23900241 10.1038/ncomms3192 PMC3736576

[12] Strong R, Miller RA, Antebi A, Astle CM, Bogue M, Denzel MS, et al. Longer lifespan in male mice treated with a weakly estrogenic agonist, an antioxidant, an α-glucosidase inhibitor or a Nrf2-inducer. Aging Cell. 2016;15(5):872–84. 10.1111/acel.12496. 27312235 10.1111/acel.12496 PMC5013015

[13] Harrison DE, Strong R, Allison DB, Ames BN, Astle CM, Atamna H, et al. Acarbose, 17-α-estradiol, and nordihydroguaiaretic acid extend mouse lifespan preferentially in males. Aging Cell. 2014;13(2):273–82. 10.1111/acel.12170. 24245565 10.1111/acel.12170 PMC3954939

[14] Miller RA, Harrison DE, Allison DB, Bogue M, Debarba L, Diaz V, et al. Canagliflozin extends life span in genetically heterogeneous male but not female mice. JCI Insight. 2020. 10.1172/jci.insight.140019. 32990681 10.1172/jci.insight.140019 PMC7710304

[15] Fang EF, Hou Y, Lautrup S, Jensen MB, Yang B, SenGupta T, et al. NAD+ augmentation restores mitophagy and limits accelerated aging in Werner syndrome. Nat Commun. 2019;10(1):5284. 10.1038/s41467-019-13172-8. 31754102 10.1038/s41467-019-13172-8 PMC6872719

[16] Kirkland JL, Tchkonia T, Zhu Y, Niedernhofer LJ, Robbins PD. The clinical potential of senolytic drugs. J Am Geriatr Soc. 2017;65(10):2297–301. 10.1111/jgs.14969. 28869295 10.1111/jgs.14969 PMC5641223

[17] Xu Q, Fu Q, Li Z, Liu H, Wang Y, Lin X, et al. The flavonoid procyanidin C1 has senotherapeutic activity and increases lifespan in mice. Nat Metab. 2021;3(12):1706–26. 10.1038/s42255-021-00491-8. 34873338 10.1038/s42255-021-00491-8 PMC8688144

[18] Yousefzadeh MJ, Zhu Y, McGowan SJ, Angelini L, Fuhrmann-Stroissnigg H, Xu M, et al. Fisetin is a senotherapeutic that extends health and lifespan. EBioMedicine. 2018;36:18–28. 10.1016/j.ebiom.2018.09.015. 30279143 10.1016/j.ebiom.2018.09.015 PMC6197652

[19] Xu M, Pirtskhalava T, Farr JN, Weigand BM, Palmer AK, Weivoda MM, et al. Senolytics improve physical function and increase lifespan in old age. Nat Med. 2018;24(8):1246–56. 10.1038/s41591-018-0092-9. 29988130 10.1038/s41591-018-0092-9 PMC6082705

[20] Tain LS, Jain C, Nespital T, Froehlich J, Hinze Y, Grönke S, et al. Longevity in response to lowered insulin signaling requires glycine N-methyltransferase-dependent spermidine production. Aging Cell. 2020;19(1):e13043. 10.1111/acel.13043. 31721422 10.1111/acel.13043 PMC6974722

[21] Eisenberg T, Knauer H, Schauer A, Büttner S, Ruckenstuhl C, Carmona-Gutierrez D, et al. Induction of autophagy by spermidine promotes longevity. Nat Cell Biol. 2009;11(11):1305–14. 10.1038/ncb1975. 19801973 10.1038/ncb1975

[22] Doeppner TR, Coman C, Burdusel D, Ancuta D-L, Brockmeier U, Pirici DN, et al. Long-term treatment with chloroquine increases lifespan in middle-aged male mice possibly via autophagy modulation, proteasome inhibition and glycogen metabolism. Aging. 2022;14(10):4195–210. 10.18632/aging.204069. 35609021 10.18632/aging.204069 PMC9186778

[23] Li W, Zou Z, Cai Y, Yang K, Wang S, Liu Z, et al. Low-dose chloroquine treatment extends the lifespan of aged rats. Protein Cell. 2022;13(6):454–61. 10.1007/s13238-021-00903-1. 35023015 10.1007/s13238-021-00903-1 PMC9095792

[24] Alle Q, Le Borgne E, Bensadoun P, Lemey C, Béchir N, Gabanou M, et al. A single short reprogramming early in life initiates and propagates an epigenetically related mechanism improving fitness and promoting an increased healthy lifespan. Aging Cell. 2022;21(11):e13714. 10.1111/acel.13714. 36251933 10.1111/acel.13714 PMC9649606

[25] Fischer KE, Hoffman JM, Sloane LB, Gelfond JA, Soto VY, Richardson AG, et al. A cross-sectional study of male and female C57BL/6Nia mice suggests lifespan and healthspan are not necessarily correlated. Aging. 2016;8(10):2370. 10.18632/aging.101059. 27705904 10.18632/aging.101059 PMC5115894

[26] Garmany A, Terzic A. Global healthspan-lifespan gaps among 183 World Health Organization member states. JAMA Netw Open. 2024;7(12):e2450241-e. 10.1001/jamanetworkopen.2024.50241. 39661386 10.1001/jamanetworkopen.2024.50241 PMC11635540

[27] Manton KG, Gu X, Lowrimore GR. Cohort changes in active life expectancy in the US elderly population: experience from the 1982–2004 national long-term care survey. J Gerontol B Psychol Sci Soc Sci. 2008;63(5):S269–81. 10.1093/geronb/63.5.S269. 18818447 10.1093/geronb/63.5.s269

[28] Cai L, Lubitz J. Was there compression of disability for older Americans from 1992 to 2003? Demography. 2007;44(3):479–95. 10.1353/dem.2007.0022. 17913007 10.1353/dem.2007.0022

[29] Allen NB, Zhao L, Liu L, Daviglus M, Liu K, Fries J, et al. Favorable cardiovascular health, compression of morbidity, and healthcare costs. Circulation. 2017;135(18):1693–701. 10.1161/CIRCULATIONAHA.116.026252. 28461414 10.1161/CIRCULATIONAHA.116.026252 PMC5476215

[30] Zhang YS, Saito Y, Crimmins EM. Changing impact of obesity on active life expectancy of older Americans. J Gerontol A Biol Sci Med Sci. 2019;74(12):1944–51. 10.1093/gerona/glz133. 31120111 10.1093/gerona/glz133 PMC6853657

[31] Beltrán-Sánchez H, Jiménez MP, Subramanian S. Assessing morbidity compression in two cohorts from the Health and Retirement Study. J Epidemiol Community Health. 2016;70(10):1011–6. 10.1136/jech-2015-206722. 27103663 10.1136/jech-2015-206722 PMC5486403

[32] Tetzlaff J, Muschik D, Epping J, Eberhard S, Geyer S. Expansion or compression of multimorbidity? 10-year development of life years spent in multimorbidity based on health insurance claims data of Lower Saxony, Germany. Int J Public Health. 2017;62(6):679–86. 10.1007/s00038-017-0962-9. 28283685 10.1007/s00038-017-0962-9

[33] Colin S, Lidia L, Bernard CC. Evaluating compression or expansion of morbidity in Canada: trends in life expectancy and health-adjusted life expectancy from 1994 to 2010. Health Promot Chronic Dis Prev Can. 2017;37(3):68. 10.24095/hpcdp.37.3.02. 10.24095/hpcdp.37.3.02 PMC5602161 28273034

[34] Crimmins EM, Hayward MD, Hagedorn A, Saito Y, Brouard N. Change in disability-free life expectancy for Americans 70 years old and older. Demography. 2009;46:627–46. 10.1353/dem.0.0070. 19771948 10.1353/dem.0.0070 PMC2831348

[35] Crimmins EM, Beltrán-Sánchez H. Mortality and morbidity trends: is there compression of morbidity? J Gerontol B Psychol Sci Soc Sci. 2011;66(1):75–86. 10.1093/geronb/gbq088. 21135070 10.1093/geronb/gbq088 PMC3001754

[36] Teo E, Fong S, Tolwinski N, Gruber J. Drug synergy as a strategy for compression of morbidity in a *Caenorhabditis elegans* model of Alzheimer’s disease. Geroscience. 2020;42(3):849–56. 10.1007/s11357-020-00169-1. 32088829 10.1007/s11357-020-00169-1 PMC7286995

[37] Mattison JA, Roth GS, Beasley TM, Tilmont EM, Handy AM, Herbert RL, et al. Impact of caloric restriction on health and survival in rhesus monkeys from the NIA study. Nature. 2012;489(7415):318–21. 10.1038/nature11432. 22932268 10.1038/nature11432 PMC3832985

[38] Cooley DM, Schlittler DL, Glickman LT, Hayek M, Waters DJ. Exceptional longevity in pet dogs is accompanied by cancer resistance and delayed onset of major diseases. J Gerontol A Biol Sci Med Sci. 2003;58(12):B1078–84. 10.1093/gerona/58.12.B1078. 14684704 10.1093/gerona/58.12.b1078

[39] Bansal A, Zhu LJ, Yen K, Tissenbaum HA. Uncoupling lifespan and healthspan in *Caenorhabditis elegans* longevity mutants. Proc Natl Acad Sci U S A. 2015;112(3):E277-86. 10.1073/pnas.1412192112. 25561524 10.1073/pnas.1412192112 PMC4311797

[40] Statzer C, Reichert P, Dual J, Ewald CY. Longevity interventions temporally scale healthspan in *Caenorhabditis elegans*. iScience. 2022;25(3):103983. 10.1016/j.isci.2022.103983. 35310333 10.1016/j.isci.2022.103983 PMC8924689

[41] Papadopoulos NT, Papanastasiou S, Müller HG, Wang JL, Yang W, Carey JR. Dietary effects on sex-specific health dynamics of medfly: support for the dynamic equilibrium model of aging. Exp Gerontol. 2011;46(12):1026–30. 10.1016/j.exger.2011.08.013. 21933703 10.1016/j.exger.2011.08.013 PMC3382071

[42] Thapa D, Najam W, Kpormegbey D, Robertson O, Mokalla T, Becerra-Garcia L, et al. Compression of morbidity dynamics: interrogating evidence, measurement challenges, and research horizons. UAB Aging Research Symposium 2024; University of Alabama, Birmingham, AL 2024.

[43] Kaeberlein M. How healthy is the healthspan concept? Geroscience. 2018;40(4):361–4. 10.1007/s11357-018-0036-9. 30084059 10.1007/s11357-018-0036-9 PMC6136295

[44] Brown AW, Kaiser KA, Allison DB. Issues with data and analyses: errors, underlying themes, and potential solutions. Proc Natl Acad Sci U S A. 2018;115(11):2563–70. 10.1073/pnas.1708279115. 29531079 10.1073/pnas.1708279115 PMC5856502

[45] Yang Y, Mayo A, Levy T, Raz N, Shenhar B, Jarosz DF, et al. Compression of morbidity by interventions that steepen the survival curve. Nat Commun. 2025;16(1):3340. 10.1038/s41467-025-57807-5. 40199852 10.1038/s41467-025-57807-5 PMC11978790

[46] Lamming DW. Quantification of healthspan in aging mice: introducing FAMY and GRAIL. Geroscience. 2024;46(5):4203–15. 10.1007/s11357-024-01200-5. 38755467 10.1007/s11357-024-01200-5 PMC11336093

[47] Woo J, Zheng Z, Leung J, Chan P. Prevalence of frailty and contributory factors in three Chinese populations with different socioeconomic and healthcare characteristics. BMC Geriatr. 2015;15(1):163. 10.1186/s12877-015-0160-7. 26652647 10.1186/s12877-015-0160-7 PMC4675032

[48] Yu R, Wu W-C, Leung J, Hu SC, Woo J. Frailty and its contributory factors in older adults: a comparison of two Asian regions (Hong Kong and Taiwan). Int J Environ Res Public Health. 2017;14(10):1096. 10.3390/ijerph14101096. 28934150 10.3390/ijerph14101096 PMC5664597

[49] Di Francesco A, Deighan AG, Litichevskiy L, Chen Z, Luciano A, Robinson L, et al. Dietary restriction impacts health and lifespan of genetically diverse mice. Nature. 2024;634(8034):684–92. 10.1038/s41586-024-08026-3. 39385029 10.1038/s41586-024-08026-3 PMC11485257

[50] Green CL, Trautman ME, Chaiyakul K, Jain R, Alam YH, Babygirija R, et al. Dietary restriction of isoleucine increases healthspan and lifespan of genetically heterogeneous mice. Cell Metab. 2023;35(11):1976–95.e6. 10.1016/j.cmet.2023.10.005. 37939658 10.1016/j.cmet.2023.10.005 PMC10655617

[51] Shahmirzadi AA, Edgar D, Liao C-Y, Hsu Y-M, Lucanic M, Asadi Shahmirzadi A, et al. Alpha-ketoglutarate, an endogenous metabolite, extends lifespan and compresses morbidity in aging mice. Cell Metab. 2020;32(3):447–56.e6. 10.1016/j.cmet.2020.08.004. 32877690 10.1016/j.cmet.2020.08.004 PMC8508957

[52] Parks RJ, Fares E, MacDonald JK, Ernst MC, Sinal CJ, Rockwood K, et al. A procedure for creating a frailty index based on deficit accumulation in aging mice. J Gerontol A Biol Sci Med Sci. 2011;67A(3):217–27. 10.1093/gerona/glr193. 10.1093/gerona/glr193 22021390

[53] Whitehead JC, Hildebrand BA, Sun M, Rockwood MR, Rose RA, Rockwood K, et al. A clinical frailty index in aging mice: comparisons with frailty index data in humans. J Gerontol A Biol Sci Med Sci. 2014;69(6):621–32. 10.1093/gerona/glt136. 24051346 10.1093/gerona/glt136 PMC4022099

[54] Rockwood K, Blodgett JM, Theou O, Sun MH, Feridooni HA, Mitnitski A, et al. A frailty index based on deficit accumulation quantifies mortality risk in humans and in mice. Sci Rep. 2017;7(1):43068. 10.1038/srep43068. 28220898 10.1038/srep43068 PMC5318852

[55] Fries JF. Aging, natural death, and the compression of morbidity. N Engl J Med. 1980;303(3):130–5. 10.1056/nejm198007173030304. 7383070 10.1056/NEJM198007173030304

[56] Olshansky SJ, Rudberg MA, Carnes BA, Cassel CK, Brody JA. Trading off longer life for worsening health: the expansion of morbidity hypothesis. J Aging Health. 1991;3(2):194–216. 10.1177/089826439100300205.

[57] Gruenberg EM. The failures of success. Milbank Q. 1977;55(1):3–24. 10.1111/j.1468-0009.2005.00400.x. 141009

[58] Cox LS, Mattison JA. Increasing longevity through caloric restriction or rapamycin feeding in mammals: common mechanisms for common outcomes? Aging Cell. 2009;8(5):607–13. 10.1111/j.1474-9726.2009.00509.x. 19678809 10.1111/j.1474-9726.2009.00509.x PMC5144999

[59] Scott AJ, Ellison M, Sinclair DA. The economic value of targeting aging. Nat Aging. 2021;1(7):616–23. 10.1038/s43587-021-00080-0. 37117804 10.1038/s43587-021-00080-0 PMC10154220

[60] Corder KM, Hoffman JM, Sogorovic A, Yang Y, Banerjee A, Sun Y, et al. Negative effects of lifespan extending intervention on resilience in mice. PLoS ONE. 2024;19(11):e0312440. 10.1371/journal.pone.0312440. 39570905 10.1371/journal.pone.0312440 PMC11581327

[61] Alison L, Laura R, Gaven G, Bonnie L, Ron K, Andrea Di F, et al. Longitudinal fragility phenotyping predicts lifespan and age-associated morbidity in C57BL/6 and diversity outbred mice. bioRxiv. 2024:2024.02.06.579096. 10.1101/2024.02.06.579096.

[62] Liao CY, Rikke BA, Johnson TE, Diaz V, Nelson JF. Genetic variation in the murine lifespan response to dietary restriction: from life extension to life shortening. Aging Cell. 2010;9(1):92–5. 10.1111/j.1474-9726.2009.00533.x. 19878144 10.1111/j.1474-9726.2009.00533.x PMC3476836

[63] Parker ES, Golzarri-Arroyo L, Dickinson S, Henschel B, Becerra-Garcia L-E, Mokalla TR, et al. Improving statistical rigor in animal aging research by addressing clustering and nesting effects: illustration with the National Institute on Aging’s Intervention Testing Program Data. 2025:bioRxiv 2025.03.14.642436. 10.1101/2025.03.14.642436. 10.1093/gerona/glag074 PMC13070610 41837372

[64] Carnes BA, Holden LR, Olshansky SJ, Witten MT, Siegel JS. Mortality partitions and their relevance to research on senescence. Biogerontology. 2006;7(4):183–98. 10.1007/s10522-006-9020-3. 16732401 10.1007/s10522-006-9020-3

[65] El-Gohary A, Alshamrani A, Al-Otaibi AN. The generalized Gompertz distribution. Appl Math Model. 2013;37(1):13–24. 10.1016/j.apm.2011.05.017.

[66] Ergin A, Muntner P, Sherwin R, He J. Secular trends in cardiovascular disease mortality, incidence, and case fatality rates in adults in the United States. Am J Med. 2004;117(4):219–27. 10.1016/j.amjmed.2004.03.017. 15308430 10.1016/j.amjmed.2004.03.017

[67] Gelman A, Stern H. The difference between “significant” and “not significant” is not itself statistically significant. Am Stat. 2006;60(4):328–31. 10.1198/000313006X152649.

