# Supplementary material for: Life-extending interventions do not necessarily result in compression of morbidity: a case example offering a robust statistical approach
Source: GeroScience. 2025 Nov 12;48(1):263–81. doi: 10.1007/s11357-025-01925-x (PMC12972480; doi:10.1007/s11357-025-01925-x)
Supplement: Supplementary file 1 — Supplementary Material 1 (DOCX 42.0 KB) [file 11357_2025_1925_MOESM1_ESM.docx]

**Supplementary material 1**

**Supplementary Table 1** Difference between rate of decline in vitality and survival, by intervention group (within-group analysis) – difference calculated on actual days of frailty (vitality) measurement

|  | **Within-group analysis** |  |  | **Paired t-test** | | | | | **Wilcoxon signed-rank test** | |
| --- | --- | --- | --- | --- | --- | --- | --- | --- | --- | --- |
|  | **Variables** | ***n**** | **Mean** | **Mean difference (Vitality – Survival)** | **SE** | ***P*-value** | **Bootstrapped SE** | **Bootstrapped *P*-value** | ***P*- value** | **Bootstrapped *P*-value** |
| **Di Francesco et al. [49] – Female mice** | | | | | | | | | | |
| AL (Control) | Rate of decline in vitality | 5 | 0.00027 | -0.00047 | 0.00038 | 0.284 | 0.00034 | 0.166 | 0.438 | 0.294 |
|  | Rate of decline in survival | 5 | 0.00074 |  |  |  |  |  |  |  |
| 1D IF | Rate of decline in vitality | 5 | 0.00020 | -0.00043 | 0.00032 | 0.259 | 0.00029 | 0.139 | 0.438 | 0.294 |
|  | Rate of decline in survival | 5 | 0.00063 |  |  |  |  |  |  |  |
| 2D IF | Rate of decline in vitality | 5 | 0.00016 | -0.00039 | 0.00029 | 0.246 | 0.00026 | 0.125 | 0.313 | 0.142 |
|  | Rate of decline in survival | 5 | 0.00056 |  |  |  |  |  |  |  |
| 20% CR | Rate of decline in vitality | 5 | 0.00017 | -0.00028 | 0.00024 | 0.307 | 0.00021 | 0.186 | 0.625 | 0.488 |
|  | Rate of decline in survival | 5 | 0.00045 |  |  |  |  |  |  |  |
| 40% CR | Rate of decline in vitality | 5 | 0.00016 | -0.00014 | 0.00017 | 0.460 | 0.00015 | 0.355 | 0.625 | 0.488 |
|  | Rate of decline in survival | 5 | 0.00030 |  |  |  |  |  |  |  |
| **Green et al. [50] – Female mice** | | | | | | | | | | |
| Control AA | Rate of decline in vitality | 32 | 0.00022 | -0.00211 | 0.00070 | 0.005 | 0.00071 | 0.003 | 0.038 | 0.031 |
|  | Rate of decline in survival | 32 | 0.00233 |  |  |  |  |  |  |  |
| Low AA | Rate of decline in vitality | 42 | 0.00032 | -0.00070 | 0.00029 | 0.021 | 0.00029 | 0.014 | 0.365 | 0.353 |
|  | Rate of decline in survival | 42 | 0.00103 |  |  |  |  |  |  |  |
| Low Ile | Rate of decline in vitality | 42 | 0.00035 | -0.00094 | 0.00042 | 0.031 | 0.00040 | 0.019 | 0.720 | 0.717 |
|  | Rate of decline in survival | 42 | 0.00129 |  |  |  |  |  |  |  |
| **Green et al. [50] – Male mice** | | | | | | | | | | |
| Control AA | Rate of decline in vitality | 51 | 0.00038 | -0.00076 | 0.00041 | 0.069 | 0.00040 | 0.057 | 0.465 | 0.481 |
|  | Rate of decline in survival | 51 | 0.00114 |  |  |  |  |  |  |  |
| Low AA | Rate of decline in vitality | 57 | 0.00038 | -0.00094 | 0.00044 | 0.039 | 0.00044 | 0.035 | 0.197 | 0.245 |
|  | Rate of decline in survival | 57 | 0.00132 |  |  |  |  |  |  |  |
| Low Ile | Rate of decline in vitality | 67 | 0.00035 | -0.00056 | 0.00039 | 0.153 | 0.00037 | 0.135 | 0.126 | 0.167 |
|  | Rate of decline in survival | 67 | 0.00091 |  |  |  |  |  |  |  |
| **Shahmirzadi et al. [51] – Female mice** | | | | | | | | | | |
| Control | Rate of decline in vitality | 20 | 0.00052 | -0.00067 | 0.00037 | 0.083 | 0.00036 | 0.065 | 0.349 | 0.351 |
|  | Rate of decline in survival | 20 | 0.00119 |  |  |  |  |  |  |  |
| Treatment (AKG) | Rate of decline in vitality | 20 | 0.00026 | -0.00074 | 0.00033 | 0.036 | 0.00033 | 0.024 | 0.189 | 0.178 |
|  | Rate of decline in survival | 20 | 0.00100 |  |  |  |  |  |  |  |
| **Shahmirzadi et al. [51] – Male mice** | | | | | | | | | | |
| Control | Rate of decline in vitality | 7 | 0.00034 | -0.00082 | 0.00059 | 0.214 | 0.00053 | 0.120 | 0.219 | 0.078 |
|  | Rate of decline in survival | 7 | 0.00117 |  |  |  |  |  |  |  |
| Treatment (AKG) | Rate of decline in vitality | 7 | 0.00018 | -0.00068 | 0.00047 | 0.194 | 0.00042 | 0.103 | 0.219 | 0.078 |
|  | Rate of decline in survival | 7 | 0.00086 |  |  |  |  |  |  |  |

**n* refers to the number of measurement points (ages) at which the vitality and survival were estimated.

Abbreviations: AA, amino acid; AL, ad libitum; 20% CR, caloric restriction at 20%; 40% CR, caloric restriction at 40%; Ile, isoleucine; 1D IF, intermittent fasting 1 day per week; 2D IF, intermittent fasting 2 consecutive days per week.

Supplementary Table 2 Difference-in-difference (between-group) analysis of the rate of decline in vitality and survival – difference calculated on actual days of frailty (vitality) measurement

| **Sample** | **Group** | ***n**** | **Mean difference**  **(Vitality – Survival)** | **One-way ANOVA** | | **Kruskal‒Wallis** | | **Linear Regression** | | | |
| --- | --- | --- | --- | --- | --- | --- | --- | --- | --- | --- | --- |
|  |  |  |  | **F statistic and *P*-value** | **Bootstrapped *P*-value** | **Chi^2^** | ***P*-value** | **Mean difference-in-difference (Treatment - Control)** | **SE of the mean difference** | ***P*-value** | **Bootstrapped *P*-value** |
| **Di Francesco et al. [49]** | | | | | | | | | | | |
| Female | Control (AL) | 5 | -0.00047 | F=0.21, *P* = 0.931 | 0.863 | 0.236 | 0.994 | Ref |  |  |  |
|  | 1D IF | 5 | -0.00043 |  |  |  |  | 0.00005 | 0.00050 | 0.928 | 0.927 |
|  | 2D IF | 5 | -0.00039 |  |  |  |  | 0.00008 | 0.00048 | 0.874 | 0.875 |
|  | 20% CR | 5 | -0.00028 |  |  |  |  | 0.00019 | 0.00045 | 0.683 | 0.684 |
|  | 40% CR | 5 | -0.00014 |  |  |  |  | 0.00033 | 0.00042 | 0.438 | 0.433 |
| **Green et al. [50]** | | | | | | | | | | | |
| Female | Control AA | 32 | -0.00211 | F=2.40, *P* = 0.095 | 0.375 | 4.745 | 0.093 | Ref |  |  |  |
|  | Low AA | 42 | -0.00070 |  |  |  |  | 0.00141 | 0.00076 | 0.065 | 0.065 |
|  | Low Ile | 42 | -0.00094 |  |  |  |  | 0.00117 | 0.00081 | 0.152 | 0.159 |
| Male | Control AA | 51 | -0.00076 | F=0.22, *P* = 0.802 | 0.864 | 1.515 | 0.469 | Ref |  |  |  |
|  | Low AA | 57 | -0.00094 |  |  |  |  | -0.00018 | 0.00060 | 0.770 | 0.760 |
|  | Low Ile | 67 | -0.00056 |  |  |  |  | 0.00020 | 0.00056 | 0.722 | 0.715 |
| **Shahmirzadi et al. [51]** | | | | | | | | | | | |
|  |  |  |  | **Independent sample *t*-test** | | | | | **Wilcoxon rank-sum test** | |  |
|  |  |  | **Mean (Difference)** | **Difference-in-difference** | **SE** | ***P*-value** | **Bootstrapped SE** | **Bootstrapped *P*-value** | ***P*-value** | **Bootstrapped *P*-value** |  |
| Female | Treatment | 20 | -0.00074 | -0.00007 | 0.00049 | 0.894 | 0.00048 | 0.890 | 0.583 | 0.585 |  |
|  | Control | 20 | -0.00067 |  |  |  |  |  |  |  |  |
| Male | Treatment | 7 | -0.00068 | 0.00014 | 0.00076 | 0.856 | 0.00074 | 0.849 | 0.902 | 0.863 |  |
|  | Control | 7 | -0.00082 |  |  |  |  |  |  |  |  |

**n* refers to the number of measurement points (ages) at which the vitality and survival were estimated.

Abbreviations: AA, amino acid; AL, ad libitum; 20% CR, caloric restriction at 20%; 40% CR, caloric restriction at 40%; Ile, isoleucine; 1D IF, intermittent fasting 1 day per week; 2D IF, intermittent fasting 2 consecutive days per week.

Supplementary Table 3 Difference-in-difference (between-group) analysis of the rate of decline in vitality and survival among very old mice (age days ≥ 1050 days)

| **Sample** | **Group** | ***n**** | **Mean difference (Vitality – Survival)** | **One-way ANOVA** | | **Kruskal‒Wallis** | | **Linear Regression** | | | |
| --- | --- | --- | --- | --- | --- | --- | --- | --- | --- | --- | --- |
|  |  |  |  | **F statistic and *P*-value** | **Bootstrapped *P*-value** | **Chi^2^** | ***P*-value** | **Difference-in-difference (Treatment - Control)** | **SE** | ***P*-value** | **Bootstrapped *P*-value** |
| **Di Francesco et al. [49]** | | | | | | | | | | | |
| Female | Control (AL) | 9 | -0.00097 | F= 1.14, *P* = 0.350 | 0.495 | 5.20 | 0.267 | Ref |  |  |  |
|  | 1D IF | 7 | -0.00127 |  |  |  |  | -0.00031 | 0.00021 | 0.148 | 0.144 |
|  | 2D IF | 11 | -0.00107 |  |  |  |  | -0.00010 | 0.00020 | 0.605 | 0.609 |
|  | 20% CR | 14 | -0.00093 |  |  |  |  | -0.00004 | 0.00019 | 0.833 | 0.838 |
|  | 40% CR | 18 | -0.00094 |  |  |  |  | -0.00003 | 0.00018 | 0.873 | 0.874 |
| **Green et al. [50]** | | | | | | | | | | | |
| Female | Control AA | 2 | -0.00081 | F=0.55, *P* = 0.598 | 0.890 | 1.09 | 0.580 | Ref |  |  |  |
|  | Low AA | 5 | -0.00170 |  |  |  |  | -0.00090 | 0.00062 | 0.194 | 0.013 |
|  | Low Ile | 3 | -0.00154 |  |  |  |  | -0.00073 | 0.00079 | 0.387 | 0.069 |
| Male | Control AA | 4 | -0.00147 | F=0.31, *P* = 0.739 | 0.895 | 0.30 | 0.862 | Ref |  |  |  |
|  | Low AA | 3 | -0.00145 |  |  |  |  | 0.00002 | 0.00053 | 0.978 | 0.772 |
|  | Low Ile | 6 | -0.00193 |  |  |  |  | -0.00046 | 0.00065 | 0.497 | 0.276 |
| **Shahmirzadi et al. [51]** | | | | | | | | | | | |
|  |  |  |  | **Independent sample *t*-test** | | | | | **Wilcoxon rank-sum test** | |  |
|  |  |  | **Mean (Difference)** | **Difference-in-difference** | **SE** | ***P*-value** | **Bootstrapped SE** | **Bootstrapped *P*-value** | ***P*-value** | **Bootstrapped *P*-value** |  |
| Female | Treatment | 1 |  |  |  |  |  |  |  |  |  |
|  | Control | 0 |  |  |  |  |  |  |  |  |  |
| Male | Treatment | 6 | -0.00283 | -0.00032 | 0.00146 | 0.832 | 0.00144 | 0.825 | 0.610 | 0.525 |  |
|  | Control | 4 | -0.00251 |  |  |  |  |  |  |  |  |

**n* refers to the number of measurement points (ages) at which the vitality and survival were estimated. None of the mice in control group in female mice in Shahmirzadi et al. [50] survived beyond 1050 days.

Abbreviations: AA, amino acid; AL, ad libitum; 20% CR, caloric restriction at 20%; 40% CR, caloric restriction at 40%; Ile, isoleucine; 1D IF, intermittent fasting 1 day per week; 2D IF, intermittent fasting 2 consecutive days per week.
